# Heart immunoengineering by lentiviral vector-mediated genetic modification during normothermic *ex vivo* perfusion

**DOI:** 10.3389/fimmu.2024.1404668

**Published:** 2024-06-05

**Authors:** Katharina Schmalkuche, Tamina Rother, Jonathan M. Burgmann, Henrike Voß, Klaus Höffler, Günes Dogan, Arjang Ruhparwar, Jan D. Schmitto, Rainer Blasczyk, Constanca Figueiredo

**Affiliations:** ^1^Institute of Transfusion Medicine and Transplant Engineering, Hannover Medical School, Hannover, Germany; ^2^Transregional Collaborative Research Centre 127, Hannover Medical School, Hannover, Germany; ^3^Department of Cardiothoracic, Transplantation, and Vascular Surgery, Hannover Medical School, Hannover, Germany

**Keywords:** heart immunoengineering, genetic modification, lentiviral vector, normothermic *ex vivo* perfusion, transplantation

## Abstract

Heart transplantation is associated with major hurdles, including the limited number of available organs for transplantation, the risk of rejection due to genetic discrepancies, and the burden of immunosuppression. In this study, we demonstrated the feasibility of permanent genetic engineering of the heart during *ex vivo* perfusion. Lentiviral vectors encoding for short hairpin RNAs targeting beta2-microglobulin (shβ2m) and class II transactivator (shCIITA) were delivered to the graft during two hours of normothermic EVHP. Highly efficient genetic engineering was indicated by stable reporter gene expression in endothelial cells and cardiomyocytes. Remarkably, swine leucocyte antigen (SLA) class I and SLA class II expression levels were decreased by 66% and 76%, respectively, in the vascular endothelium. Evaluation of lactate, troponin T, and LDH levels in the perfusate and histological analysis showed no additional cell injury or tissue damage caused by lentiviral vectors. Moreover, cytokine secretion profiles (IL-6, IL-8, and TNF-α) of non-transduced and lentiviral vector-transduced hearts were comparable. This study demonstrated the *ex vivo* generation of genetically engineered hearts without compromising tissue integrity. Downregulation of SLA expression may contribute to reduce the immunogenicity of the heart and support graft survival after allogeneic or xenogeneic transplantation.

## 1 Introduction

Heart transplantation (HTx) remains the gold standard therapy to prolong the lives of patients with end-stage heart failure or chronic diseases caused by incurable heart dysfunctions (1). Since the first heart transplantation in 1967, significant progress has been made in the development of mechanical circulatory support devices as a bridge to transplantation, efficient organ allocation networks, and optimized immunosuppressive regimens, contributing to increased graft survival up to 14.8 years (2, 3). Nevertheless, acute cellular rejection caused by allogeneic T cell-mediated responses and donor-specific antibodies targeting mismatched human leukocyte antigens (HLA) class I and II proteins remain the major immunological causes for graft failure after HTx. In addition, cardiac allograft vasculopathy (CAV), which contributes significantly to the mortality of HTx patients, is mediated by immunopathological mechanisms leading to remodeling of the allograft vasculature. After HTx, CAV is induced by injury of the endothelial cell (EC) layer and stimulation of smooth cell proliferation due to ischemia-reperfusion injury or allogeneic immune responses (4, 5). Hence, HLA mismatches between donor and recipient remain a major cause of premature organ dysfunction and reduced allograft survival (6). Similarly, swine leukocyte antigens (SLA) expressed on porcine organs might be recognized by pre-formed or *de novo* xenoreactive T cells after xenotransplantation. Furthermore, SLA expression facilitates the presentation of the wide panel of discrepant minor histocompatibility antigens between humans and pigs, thereby increasing the probability of T cell-mediated responses and xenograft rejection. Previously, we have shown that downregulation of HLA or SLA expression decreases cell immunogenicity and reduces the strength of immune responses in allogeneic and xenogeneic settings, respectively.

Recently, the heart has become a major target for gene therapy to prevent heart failure. Beneficial effects of VEGF-A, VEGF-B, or SDF-1 gene therapy have been described in pre-clinical trials. These approaches were based on the application of adenoviral vectors or adeno-associated viral (AAV) vectors by arterial and venous infusion, direct myocardial injection, and pericardial injection as administration routes, resulting in localized genetic modification of the cardiac tissue (7–9). So far, lentiviral vectors have been less exploited due to their poor stability and complex manufacturing process, yielding low titers. However, lentiviral vectors mediate the permanent expression of delivered transgenes (10–12).

*Ex vivo* genetic engineering of organs has emerged as an approach to minimize safety concerns by eliminating the risk of off-target effects associated with the transduction of non-targeted organs or tissues after systemic administration. However, the preservation time of donor hearts is typically shorter than that of other organs, as the cardiac tissue cannot tolerate prolonged ischemia. Normothermic *ex vivo* organ perfusion has been established as an alternative to static cold storage, limiting ischemic time and allowing assessment of graft function (13). Remarkably, *ex vivo* perfusion represents a suitable route for the delivery of drugs or viral vectors, enabling their re-circulation through the organ and maximizing their effect. Previously, we demonstrated that *ex vivo* organ perfusion allowed efficient lentiviral vector transduction of pig lungs, rat kidneys, and limbs (14–16).

Therefore, in this study, we aimed to establish a method for genetically engineering the cardiac tissue along the entire heart in a stable manner to reduce SLA expression. The development of strategies to permanently genetically engineer the heart may provide a powerful tool to correct various subsets of genetic disorders such as inherited arrhythmias, cardiomyopathies, vascular diseases, and structural heart defects as well as decrease heart immunogenicity to support graft survival after allogeneic or xenogeneic transplantation.

## 2 Materials and methods

### 2.1 Lentiviral vector constructs and production

The lentiviral vector plasmid pRRL.PPT.eFS.pre was used to clone an RNAi cassette consisting of U6 and H1 promoter sequences that regulate the expression of short hairpin RNAs (shRNAs) targeting swine beta2-microglobulin (β2m) (shβ2m: 5’-CGCGTCCCCGCACGTGACTCTCGATAAGCCTTCAAGAGAGGCTTATCGAGAGTCACGTGCTTTTTGGAAAT-3’) and swine class II transactivator (CIITA) (shCIITA: 5’-CGCGTCCCCGCTGCCACAGTAC GACTTTGTTTCAAGAGAACAAAGTCGTACTGTGGCAGCTTTTTGGAAAT-3’), respectively. Consequently, the construct was designed to downregulate the expression of SLA class I and class II transcripts. A lentiviral vector plasmid encoding for two non‐specific shRNAs (shNS) sequences was used as a control. Sequences for NeonGreen or a secreted luciferase enzyme Nanoluciferase (NanoLuc) from Oplophorus gracilirostris were cloned into the lentiviral vector plasmid under the regulation of an SFFV or elongation factor 1α (EF-1α) core promoter and served as reporter genes.

Lentiviral vector particles were produced in HEK293T cells and cultured in HYPERFlask Cell Culture Vessels (Corning, Corning, NewYork, USA) using Dulbecco’s modified Eagle’s medium (DMEM; Lonza, Basel, Switzerland) supplemented with 10% fetal calf serum (FCS; Sigma-Aldrich, St. Louis, Missouri, USA), 1% glutamine (PAN-Biotech GmbH, Aidenbach, Germany) and 2% penicillin-streptomycin (Life Technologies, Carlsbad, California, USA). After reaching 80–90% confluence, HEK293T cells were co-transfected with the shRNAs-encoding vector as well as psPAX2 and pMD2.G plasmids mixed with polyethylenimine (Polysciences, Inc., Warrington, Pennsylvania, USA) for 64 hours. Cell culture supernatants were collected and centrifuged for 3 hours at 20,000 g and 16°C. Lentiviral vector pellets were resuspended in Ringer’s solution (Deltamedica, Reutlingen, Germany) and viral vector titration was performed by p24 enzyme-linked immunosorbent assay (Cell Biolabs Inc., San Diego, California, USA).

### 2.2 Experimental animals and heart retrieval

Animal experiments were approved by the supervisory authority (LAVES-Niedersächsisches Landesamt für Verbraucherschutz und Lebensmittelsicherheit) according to the recommendation of their Ethics Committee and performed in compliance with the ARRIVE guidelines, the German animal welfare law, the German guidelines for animal welfare and the EU Directive 2010/63/EU.

Porcine hearts were obtained from German Landrace pigs (both sexes, mean age ± 8 months). After deep anesthesia with 25 mg/kg Zoletil (Virbac S.A., Carros, France), 0.05 mg/kg atropine (EIFELFANGO GmbH & Co. KG, Bad Neuenahr-Ahrweiler, Germany), 3 mg/kg propofol (Hikma Pharma GmbH, Martinsried, Germany), and isoflurane (Baxter Deutschland GmbH, Unterschleißheim, Germany), local anesthesia with mepivacaine (4 mg/kg) was applied. Then, median sternotomy with pericardial opening was performed and the heart was inspected for contractility of the ventricles and possible coronary artery sclerosis. The hearts were flushed anterogradely with 1 L hyperkalemic Custodiol® (Dr. Franz Köhler Chemie GmbH, Bensheim, Germany) through the vena cava and the aorta to induce cardioplegia, remove residual blood, and minimize metabolic activity. Afterwards, the pericardium was incised and the aorta, pulmonary artery, vein, and vena cava were transected proximal to the cardiac hilum. The hearts were carefully removed and placed on ice for cannulation of the aorta and pulmonary artery. Finally, the retrieved hearts were transferred to the self-assembled heart perfusion system and connected via the aortic cannula.

### 2.3 Heart genetic engineering with lentiviral vector particles during *ex vivo* heart perfusion (EVHP)

**Figure 1:**
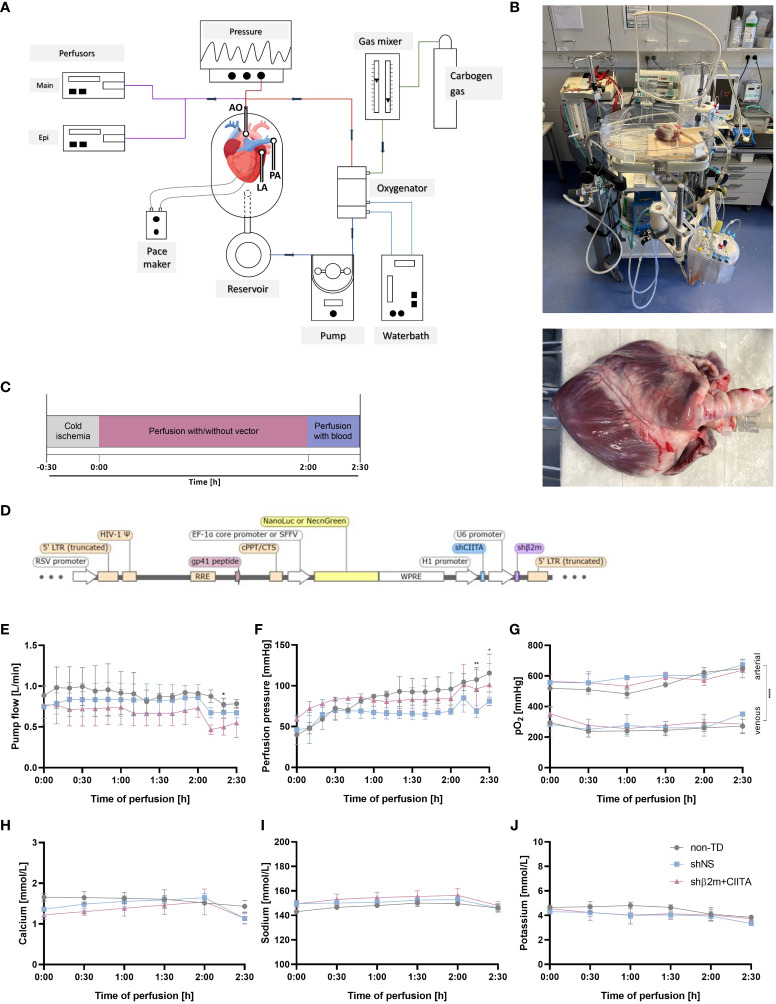
Genetic engineering of the heart during normothermic *ex vivo* perfusion. **(A)** Schematic representation of the *ex vivo* heart perfusion (EVHP) system constituted by the heart chamber, reservoir, pump unit, and oxygenator. The lines represent the oxygenated (red) and deoxygenated (blue) perfusion solution, circulating through the system. The cannulated aorta guides the perfusion solution into the branches of the coronary arteries, while the cannulated pulmonary trunk allows venous outflow (illustration was created with Biorender.com) **(B)** Representative images of the heart connected to the EVHP system. **(C)** Timeline of the process from organ retrieval to genetic engineering to the end of EVHP. **(D)** Lentiviral vector map: The lentiviral vector encodes for two short hairpin RNAs targeting swine beta2-microglobulin (shβ2m) and swine class II transactivator (shCIITA) under the control of U6 and H1 promoter sequences, respectively. Sequences for NeonGreen or a secreted Nanoluciferase (NanoLuc) were regulated by SFFV or EF-1α core promoter, respectively, and served as reporter genes. LTR, long terminal repeat; RRE, rev response element; WPRE, WHP posttranscriptional response element. **(E–G)** Comparison of pump flow, perfusion pressure, and arterial and venous oxygen partial pressures (pO_2_) between non-transduced (non-TD), shNS-transduced, and shβ2m+CIITA-transduced hearts. **(H–J)** Comparison of calcium, sodium, and potassium levels between non-TD, shNS-transduced, and shβ2m+CIITA-transduced hearts. Graphs represent mean and standard deviation. Analyzed perfusion parameters and electrolyte levels did not differ significantly between the individual groups before blood addition (*p<0.05; **p<0.01; ****p<0.0001; two-way ANOVA).

The connected hearts immediately started to beat with increasing temperature. Cardioversion was performed as required to maintain a physiologic rhythm and the heart rate was adjusted to 60–80 beats per minute. The arterial line of the heart perfusion system allowed retrograde perfusion of the organ via the coronary arteries by delivering an oxygenated perfusion solution. Thereafter, the perfusion solution was guided into the coronary veins, accumulated in the right atrium and right ventricle, and left the organ through the cannulated pulmonary artery into the heart chamber of the perfusion system. The perfusion solution was then heated to 35°C, oxygenated with carbogen (95% O_2_:5% CO_2_), and finally returned to the aorta. A schematic illustration of the normothermic perfusion circuit is shown in **Figure 1A**. Perfusion parameters such as pump flow and perfusion pressure were continuously monitored.

Heart perfusions were performed for 120 minutes using 1 L Krebs-Henseleit Buffer (Sigma-Aldrich) supplemented with 300 mL 20% human albumin (Biotest Pharma GmbH, Dreieich, Germany) to prevent edema. Furthermore, 250 mg methylprednisolone (mibe GmbH Arzneimittel, Brehna, Germany), Frekavit (Paesel & Lorei GmbH & Co. KG, Rheinberg, Germany), 1 g cefazolin (Hikma Pharma GmbH), 100 mg ciprofloxacin (Basics GmbH, Leverkusen, Germany), and 8.4% sodium bicarbonate (B. Braun, Melsungen, Germany) were added to achieve a physiological pH of 7.4. Nutrient supply was maintained by infusing 20 mL of TransMedics® Maintenance solution (TransMedics® Inc., Andover, Massachusetts, USA) per hour. In addition, a continuous adrenaline supplementation of 3 mL per hour was administered. For genetic modification of the heart, 5.0x10^12^ lentiviral vector particles were added to the circulating perfusion solution. Additional 1.0x10^12^ lentiviral vector particles were injected directly into the left atrium. 8.0 mg protamine sulfate were used as a transduction enhancer. Three hearts were perfused with lentiviral vector particles encoding for shβ2m and shCIITA or shNS, respectively. Two hearts perfused without lentiviral vector particles served as controls. After 120 minutes, the perfusion solution was replaced with fresh leukocyte-depleted autologous blood supplemented with 250 mg methylprednisolone (mibe GmbH Arzneimittel), Frekavit (Paesel & Lorei GmbH & Co. KG), 1 g cefazolin (Hikma Pharma GmbH), and 100 mg ciprofloxacin (Basics GmbH). Perfusate samples were collected every 30 minutes, centrifuged at 1,500 rpm for 10 minutes, and stored at -80°C until analysis.

### 2.4 Analysis of physiological parameters during EVHP

During EVHP, perfusate samples were collected at 30-minute intervals from the aortic root (representing arterial inflow) and pulmonary artery cannula (representing venous outflow) of the heart. Samples were analyzed for oxygen partial pressure, electrolytes (sodium, calcium, potassium), and lactate levels using commercially available i-STAT CG4+ and i-STAT CG8+ cartridges and the i-STAT Alinity Blood Analyzer (all Abbott Laboratories, Chicago, Illinois, USA).

### 2.5 Quantification of troponin T levels

Troponin T levels were analyzed in perfusate samples using the cobas® Elecsys Troponin T high sensitive assay (Roche, Basel, Switzerland). Samples were measured in duplicates by the cobas® e 801 analytical unit (Roche). A 1:10 dilution was examined when the measurement range exceeded 10,000 ng/L.

### 2.6 Determination of lactate dehydrogenase (LDH) levels

LDH activity was analyzed in perfusate samples using a colorimetric Cytotoxicity Detection Kit (Roche) according to the manufacturer’s instructions. Samples were diluted 1:2 in PBS and measured in duplicates. The absorbance at 490 nm was determined by the Synergy 2 Multi-detection microplate reader (BioTek Instruments, Inc.) and the reference wavelength at 690 nm was subtracted. Optical density (OD) units were corrected by background subtraction and the calculated ODs were used to compare LDH release levels between different groups.

### 2.7 Characterization of cytokine secretion profiles

The cytokine secretion profile during EVHP was determined using Luminex technology (Luminex Corp., Austin, Texas, USA). Briefly, levels of porcine interleukin (IL)-1α, IL-1β, IL1RA, IL-6, IL-8, IL-12, interferon-gamma (IFN-γ), and tumor necrosis factor-alpha (TNF-α) were quantified in perfusate samples by the Luminex® 100/200 analyzer (Luminex Corp.). Samples and standards were prepared according to the manufacturer’s instructions and measured in duplicates. Cytokine concentrations were calculated using Xponent software version 3.1 (Luminex Corp.).

### 2.8 Isolation and cultivation of ECs and fibroblasts

After EVHP, needle biopsies were taken from the inner heart wall of the atrium and ventricle to isolate cardiac microvascular ECs and fibroblasts. Briefly, biopsies were digested with collagenase type II (Life Technologies) at 37°C for 1.5 hours and the suspension was filtered through 100µm filters to obtain a single-cell suspension. ECs were cultured in endothelial cell growth medium (EGM-2) (Lonza) on gelatin-coated plates. Fibroblasts were cultured in DMEM (Lonza) supplemented with 10% FCS (Sigma-Aldrich) and 1% glutamine (PAN-Biotech GmbH). The medium was changed every other day. After reaching 80% confluence, cells werestimulated with IFN-γ (50 ng/mL) (R&D Systems, Minneapolis,Minnesota, USA) for 48 hours to increase SLA class I expressionand induce SLA class II expression.

### 2.9 Cultivation of cardiac tissues

Needle biopsies were taken from the inner heart wall of the atrium and ventricle, and three biopsies per region were co-cultured in one well of a 12-well tissue culture plate using EGM-2 medium (Lonza). Additionally, the coronary artery and coronary vein were dissected and fragments of 0.5 cm were cultivated in EGM-2 medium (Lonza). The medium was changed every other day.

### 2.10 Quantification of bioluminescence activity

Supernatants of cultured ECs and fibroblasts as well as cultured biopsies and vessels (coronary artery and coronary vein) were collected every other day for a period of eight days. NanoLuc activity was analyzed using the Nano-Glo® Luciferase Assay System (Promega, Madison, Wisconsin, USA) according to the manufacturer’s instructions. In particular, the assay was performed by adding an equal volume of Nano-Glo® Luciferase Assay Buffer containing the substrate furimazine to the collected supernatants. After three minutes of incubation, bioluminescence activity was determined by measuring relative luminescence units (RLU) with the Lumat LB 9507 luminometer (Berthold Technologies GmbH, Zug, Switzerland).

### 2.11 Flow cytometry analysis of cardiac-specific cells

Isolated ECs and fibroblasts were characterized by flow cytometry using anti-pCD31/PECAM-1 (377537; R&D Systems) and anti-Endoglin/CD105-AF700 (MEM-229; Novus Biologicals, Littleton, Colorado, USA), or anti-Vimentin (V9; Santa Cruz, Dallas, California, USA) antibodies, respectively. Secondary antibody staining was performed with APC-labeled anti-rat IgG antibody (Invitrogen, Waltham, Massachusetts, USA) or APC-labeled anti-mouse IgG antibody (Invitrogen). IntraPrep Permeabilization Kit (Beckman Coulter, Brea, California, USA) was applied for intracellular staining of vimentin.

SLA class I and class II expression was evaluated using SLA Class I antibody (JM1E3; Bio-Rad Laboratories, Hercules, California, USA) and SLA Class II DQ antibody (K274.3G8; Bio-Rad Laboratories), followed by secondary antibody staining with PE-labeled anti-mouse IgG1 (A85–1; BD Biosciences, Allschwill, Germany) or FITC-labeled anti-mouse IgG1 antibody (RMG1–1; BioLegend, San Diego, California, USA). Antibody concentrations and dilutions were calculated according to the manufacturer’s instructions.

Data acquisition was performed by the BD FACSCanto™ II Clinical Flow Cytometer System (Becton, Dickinson & Company, Franklin Lakes, New Jersey, USA) and results were analyzed using FlowJo software v10.6.2 (Becton, Dickinson & Company).

### 2.12 Gene expression analysis

Tissue samples of atrium and ventricle were collected after EVHP and stored in RNAlater™ Stabilization Solution (Merck, Darmstadt, Germany). Non-perfused tissue samples served as a control. Total RNA from cardiac tissues, ECs, and fibroblasts was isolated with the RNeasy Mini Kit (Qiagen, Hilden, Germany) according to the manufacturer´s instructions and reverse transcribed into cDNA using the High-Capacity cDNA Reverse Transcription Kit (Applied Biosystems, Waltham, Massachusetts, USA). Gene expression of pro-inflammatory mediators or endothelial activation markers was determined by quantitative polymerase chain reaction, analyzing transcript levels of IL-6 (Ss03384604_u1), IL-8 (Ss03392437_m1), TNF-α (Ss03391318_g1), IFN-γ (Ss03391054_m1), IL-18 (Ss03391203_m1), hypoxia-inducible factor 1-alpha (HIF-1α) (Ss00922360_g1), heat shock protein 70 (Hsp70) (Ss03387784_u1), ICAM-1 (Ss03392384_m1), and VCAM-1 (Ss03390909_m1). To evaluate the silencing effect of SLA class I and class II, transcript levels of β2m (Ss03391156_m1), CIITA (Ss06941905_g1), and SLA-DQB1 (Ss03389892_m1) were examined. GAPDH (Ss03375629_u1; all from Applied Biosystems) was used as a housekeeping gene for the normalization of mRNA levels. Data were processed by StepOnePlus software v2.3 (Applied Biosystems).

### 2.13 Histological evaluation

After EVHP, needle biopsies of the atrium and ventricle were collected, fixed in 4% paraformaldehyde solution, and embedded in paraffin. Three µm sections were stained with hematoxylin and eosin (Merck) to analyze tissue integrity using a Keyence microscope (Keyence, Itasca, Illinois, USA). Visualization of NeonGreen-expressing cardiomyocytes in cultivated heart biopsies was performed by staining the corresponding sections with an anti-mNeonGreen antibody (clone 32F6, Chromotek, Planegg, Germany). Briefly, slides were heated to 98°C in citrate buffer (pH 6.0) for 40 minutes and stained with primary antibody overnight. The next day, NeonGreen-expressing cells were detected using the Zytochem Plus HRP Polymer System (Zytomed Systems, Berlin, Germany) and the Diaminobenzidine Substrate Kit (Zytomed Systems). Counterstaining was conducted with hematoxylin (Merck KGaA), followed by analysis with a Keyence microscope.

### 2.14 Statistical analysis

Statistical analyses were performed using GraphPad Prism v8.2.0 (GraphPad Software, Inc., San Diego, California, USA), and data were presented as mean ± standard deviation. One-way ANOVA with Tukey´s multiple comparison test was applied to determine differences between the means of three or more groups. Two-way ANOVA was used for comparison of data with two independent variables among multiple groups. p-values <0.05 were considered statistically significant and defined as *p<0.05, **p<0.01, ***p<0.001, and ****p<0.0001.

## 3 Results

### 3.1 Lentiviral vectors allow genetic engineering of the heart during EVHP

Normothermic EVHP represents a promising delivery route for gene therapeutic agents such as lentiviral vectors. The ability to combine the use of viral vectors with normothermic EVHP creates an excellent opportunity to genetically engineer organs during preservation prior to HTx. In this study, we perfused porcine hearts *ex vivo* with lentiviral vectors encoding for shRNAs targeting swine β2m and swine CIITA to downregulate SLA class I and class II, respectively. Non-transduced (non-TD) hearts or hearts perfused with lentiviral vectors encoding for shNS served as controls (**Figures 1A–D**). Levels of pump flow and perfusion pressure were adapted to physiological conditions and did not differ significantly between non-TD, shNS-transduced, and shβ2m+CIITA-transduced hearts within the first two hours of EVHP. However, slight differences (*p*<0.05–0.01) were observed between individual groups after the replacement of perfusion solution with autologous blood. Pump flow remained constant during perfusion, reaching mean rates of 0.91 ± 0.01 L/min for non-TD hearts, 0.86 ± 0.16 L/min for shNS-transduced hearts, and 0.73 ± 0.12 L/min for shβ2m+CIITA-transduced hearts (**Figure 1E**). Perfusion pressure started at lower levels of 40.00 ± 2.83 mmHg for non-TD hearts, 46.00 ± 18.33 mmHg for shNS-transduced hearts, and 59.67 ± 3.22 mmHg for shβ2m+CIITA-transduced hearts and increased to 96.00 ± 19.80 mmHg, 68.67 ± 4.04 mmHg, and 84.33 ± 11.93 mmHg, respectively, after two hours of EVHP (**Figure 1F**). Additionally, arterial and venous oxygen partial pressures (pO_2_) did not change considerably during EVHP. No significant differences were found between non-TD, shNS-transduced, and shβ2m+CIITA-transduced hearts, as indicated by mean arterial pO_2_ of 553.92 ± 64.59 mmHg, 596.56 ± 48.38 mmHg, and 575.28 ± 47.43 mmHg, respectively, and mean venous pO_2_ of 258.00 ± 41.52 mmHg, 280.11 ± 49.82 mmHg, and 290.39 ± 46.60 mmHg, respectively. However, the levels of arterial pO_2_ compared to venous pO_2_ differed significantly within each group (*p*<0.0001) demonstrating substantial cardiac oxygen consumption (**Figure 1G**).

The maintenance of cardiac function depends on the physiological supply of electrolytes during EVHP (17). Electrolyte levels (calcium, sodium, potassium) in the perfusion solution remained constant during perfusion and showed no significant differences between non-TD, shNS-transduced, and shβ2m+CIITA-transduced hearts. Mean calcium levels of 1.58 ± 0.13 mmol/L (non-TD), 1.46 ± 0.20 mmol/L (shNS), and 1.34 ± 0.21 mmol/L (shβ2m+CIITA) were monitored (**Figure 1H**). Accordingly, similar sodium levels of 147.17 ± 2.70 mmol/L (non-TD), 150.28 ± 2.94 mmol/L (shNS), and 152.78 ± 4.69 mmol/L (shβ2m+CIITA) (**Figure 1I**) and potassium levels of 4.46 ± 0.42 mmol/L (non-TD), 3.97 ± 0.38 mmol/L (shNS), and 4.12 ± 0.47 mmol/L (shβ2m+CIITA) (**Figure 1J**) were measured during EVHP.

### 3.2 Lentiviral vectors do not cause additional cell injury or tissue damage during EVHP

**Figure 2:**
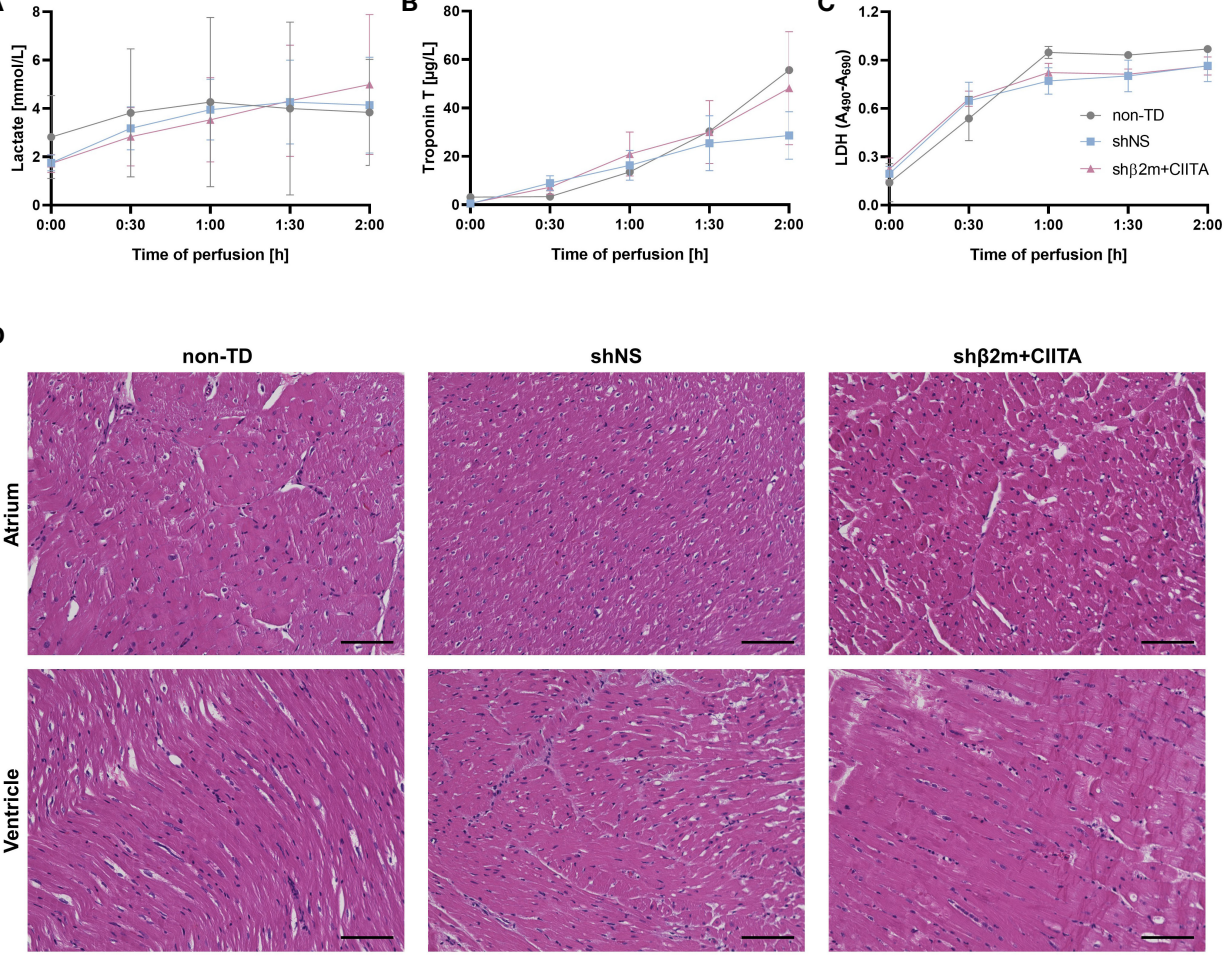
Lentiviral vector-mediated genetic engineering preserves cardiac tissue integrity. Lactate **(A)**, troponin T **(B)**, and lactate dehydrogenase (LDH) **(C)** levels were quantified in perfusates of non-TD, shNS-transduced, and shβ2m+CIITA-transduced hearts at different time points. Graphs represent mean and standard deviation of the analytes. Statistical analyses were performed using two-way ANOVA. **(D)** Histological analyses of hematoxylin and eosin-stained sections showing the atrium and ventricle of non-TD, shNS-transduced, and shβ2m+CIITA-transduced hearts (scale bar: 100 µm).

Lactate produced through anaerobic cell metabolism is considered an indicator of tissue hypoxia due to inadequate heart perfusion (18). Lactate levels in the perfusion solution increased slightly over time, starting at 2.82 ± 2.43 mmol/L for non-TD hearts, 1.75 ± 0.58 mmol/L for shNS-transduced hearts, and 1.72 ± 0.64 mmol/L for shβ2m+CIITA-transduced hearts, and reaching 3.84 ± 3.10 mmol/L, 4.14 ± 3.43 mmol/L, and 4.99 ± 5.01 mmol/L, respectively, after two hours of EVHP. Remarkably, lactate levels did not considerably change after blood addition and no significant differences were found between individual groups (**Figure 2A**).

Troponin T serves as a biomarker for the detection of myocardial damage and injury (19). Troponin T perfusate concentration increased in all groups during EVHP, reaching levels of 55.62 ± 24.32 µg/L for non-TD hearts, 28.65 ± 16.97 µg/L for shNS-transduced hearts, and 48.14 ± 40.42 µg/L for shβ2m+CIITA-transduced hearts at the end of EVHP. No significant differences were observed between individual groups (**Figure 2B**).

LDH was used as an additional tissue injury marker to estimate the potential damage induced by exposure to lentiviral vector particles during EVHP (20). LDH activity levels increased in all groups during EVHP. No significant differences were between individual groups, as indicated by OD of 0.97 ± 0.02 for non-TD hearts, 0.86 ± 0.17 for shNS-transduced hearts, and 0.86 ± 0.10 for shb2m+CIITA-transduced hearts after two hours of EVHP (**Figure 2C**).

Furthermore, histopathological examinations were performed to evaluate tissue integrity after EVHP. The atria and ventricles of non-TD, shNS-transduced, and shβ2m+CIITA-transduced hearts showed comparable findings, with mild perivascular and interstitial edema, but no evidence of necrosis, hemorrhage, or other vascular damage. Moreover, strong myocardium and no vector-specific injury were detected in all cardiac regions. Thus, evaluation of tissue injury markers and histological analysis demonstrated that genetic engineering during EVHP did not further compromise cardiac tissue integrity (**Figure 2D**).

### 3.3 Cytokine secretion profiles of non-TD and lentiviral vector-transduced hearts were comparable

**Figure 3:**
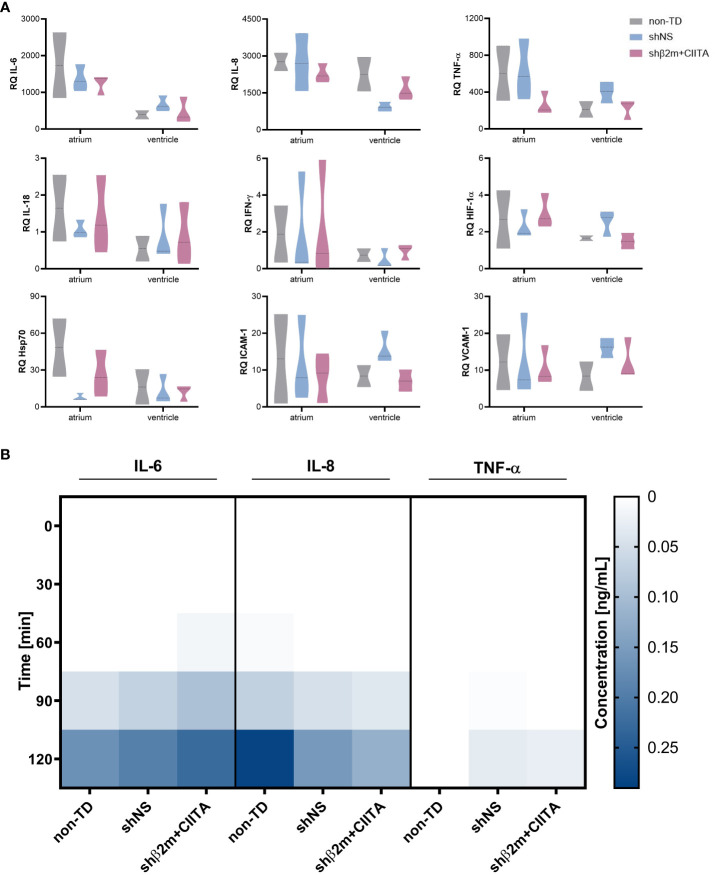
Lentiviral vector-mediated genetic engineering does not induce endothelium activation. **(A)** Relative quantification (RQ) of interleukin (IL)-6, IL-8, tumor necrosis factor-alpha (TNF-α), IL-18, interferon-gamma (IFN-γ), hypoxia-inducible factor 1-alpha (HIF-1α), heat shock protein 70 (Hsp70), intercellular adhesion molecule 1 (ICAM-1), and vascular cell adhesion molecule 1 (VCAM-1) transcript levels detected in the atrium and ventricle of non-TD, shNS-transduced, and shβ2m+CIITA-transduced hearts after EVHP. Data were normalized to the endogenous housekeeping gene GAPDH. Graphs represent mean and standard deviation. Statistical analysis was performed using two-way ANOVA. **(B)** Heatmap represents cytokine secretion of non-TD, shNS-transduced, and shβ2m+CIITA-transduced hearts during EVHP. Cytokine concentrations of IL-6, IL-8, and TNF-α were quantified in perfusates at different time points. Color saturations represent the mean of cytokine levels. Statistical analysis was performed using two-way ANOVA.

Elevated expression levels of cytokines and chemokines involved in leukocyte recruitment and activation, as well as elevated expression levels of adhesion molecules that promote extravasation of mediators into the tissue, influence transplantation outcomes by modulating donor-specific immune responses (21). In comparison to non-perfused heart tissue, transcript levels of IL-6, IL-8, and TNF-α were strongly upregulated in non-TD, shNS-transduced, and shβ2m+CIITA-transduced hearts and showing higher gene expression levels in the atrium than in the ventricle. IL-6 expression was increased 1,200- to 1,700-fold in the atrium and 400- to 700-fold in the ventricle, IL-8 expression was increased 2,300- to 2,800-fold in the atrium and 900- to 2,300-fold in the ventricle, and TNF-α expression was increased 300- to 600-fold in the atrium and 200- to 400-fold in the ventricle compared to non-perfused heart tissue. However, transcript levels did not differ significantly between individual groups or cardiac regions. While IL-18 and IFN-γ gene expression did not change considerably after EVHP, HIF-1α transcript levels were found to be 2-fold higher in the atrium and ventricle of non-TD, shNS-transduced, and shβ2m+CIITA-transduced heart tissues. The gene expression levels of intercellular adhesion molecule 1 (ICAM-1) and vascular cell adhesion molecule 1 (VCAM-1) were similar in the atrium and ventricle of non-TD, shNS-transduced, and shβ2m+CIITA-transduced heart tissues, demonstrating a 7- to 16-fold and 8- to 16-fold increase in transcript levels, respectively, compared to non-perfused heart tissue. Accordingly, Hsp70 transcripts were strongly upregulated in the atrium and ventricle of all perfused cardiac tissues (**Figure 3A**).

In particular, cytokines play a crucial role in regulating immune responses and immune cell differentiation and polarization (22). To further characterize potential changes in the inflammatory status of genetically engineered hearts, secretion levels of several pro-inflammatory cytokines were quantified. IL-1β, IL-2, IL-10, IL-18, and IFN-γ levels remained below detection limits in all groups during EVHP (data not shown). However, IL-6 and IL-8 levels increased in the perfusion solution of non-TD, shNS-transduced, and shβ2m+CIITA-transduced hearts, reaching the highest concentrations after two hours of EVHP. Remarkably, no significant differences in cytokine secretion profiles were found between individual groups. Compared to non-TD hearts (0.17 ± 0.07 ng/mL), similar IL-6 levels were measured in the perfusates of shNS-transduced (0.19 ± 0.07 ng/mL) and shβ2m+CIITA-transduced hearts (0.22 ± 0.17 ng/mL) after two hours of EVHP. A comparable cytokine release pattern was observed for IL-8 (non-TD hearts: 0.28 ± 0.28 ng/mL, shNS-transduced hearts: 0.15 ± 0.11 ng/mL, shβ2m+CIITA-transduced hearts: 0.12 ± 0.12 ng/mL). TNF-α was only detected at very low concentrations in the perfusates of shNS-transduced (0.03 ± 0.03 ng/mL) and shβ2m+CIITA-transduced hearts (0.03 ± 0.04 ng/mL) after two hours of EVHP (**Figure 3B**). These data suggest that genetic engineering did not significantly affect heart immunogenicity during EVHP.

### 3.4 EVHP with lentiviral vectors allows stable transduction of cardiac ECs and fibroblasts

**Figure 4:**
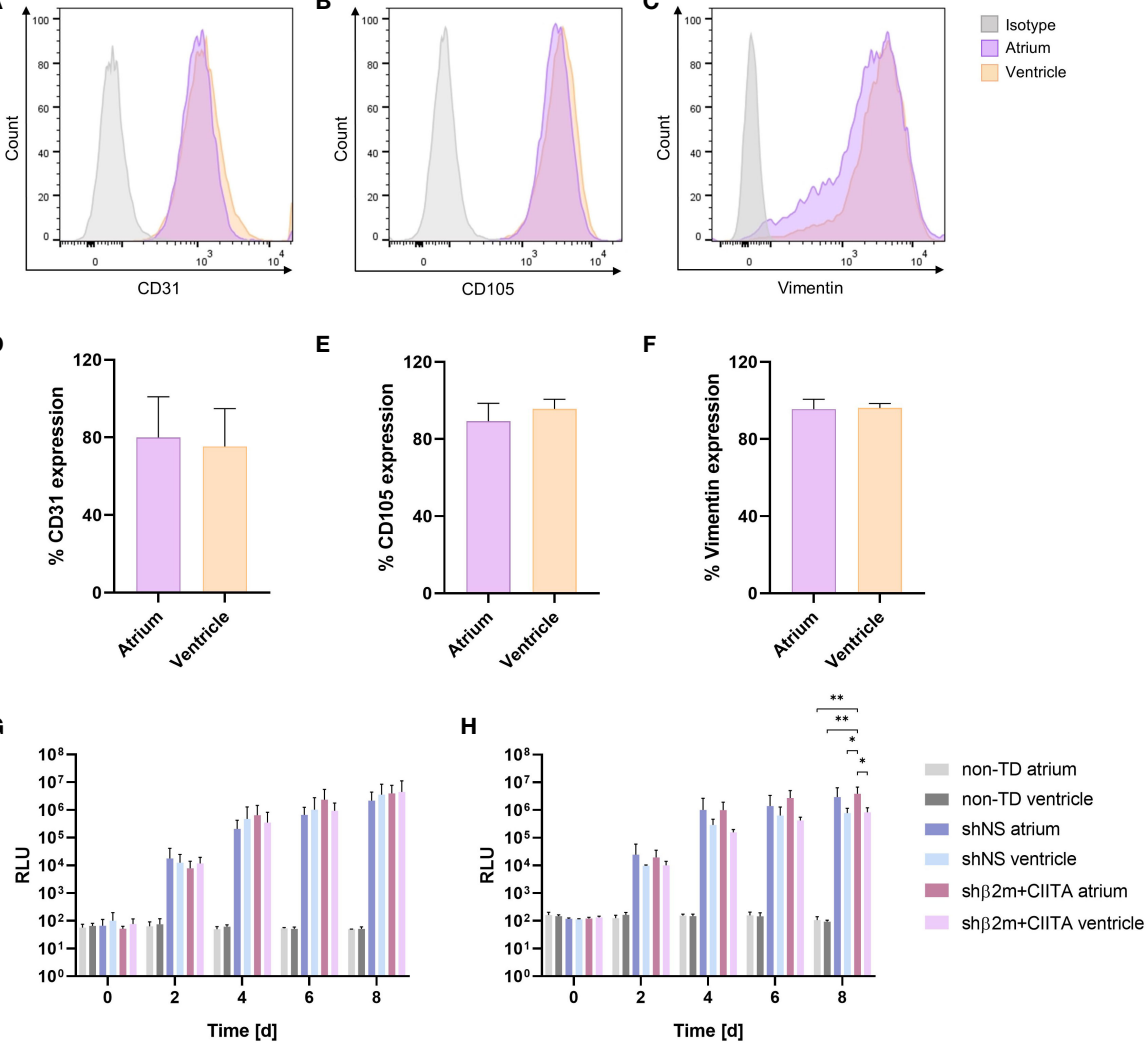
Transduction efficiency of cardiac endothelial cells (ECs) and fibroblasts. Phenotypic characterization of ECs and fibroblasts isolated from the atrium and ventricle: Figures show representative dot plots of CD31 **(A)**, CD105 **(B)**, and vimentin **(C)** expression and corresponding graphs of CD31 **(D)**, CD105 **(E)**, and vimentin **(F)** expression frequencies. Graphs represent mean and standard deviation. NanoLuc activity of ECs **(G)** and fibroblasts **(H)** isolated from the atrium and ventricle of non-TD, shNS-transduced, and shβ2m+CIITA-transduced hearts: Bioluminescence activity of cultured cells was determined by measuring relative luminescence units (RLU) over a period of eight days. Graphs represent mean and standard deviation (*p<0.05; **p<0.01; two-way ANOVA).

High transduction efficiency is essential for successful and stable genetic engineering of different heart cell types and tissues. To evaluate transduction efficiency during EVHP using lentiviral vector particles, ECs and fibroblasts were isolated from cardiac tissues. EC cultures showed high purities of CD31-positive (atrium: 79.93 ± 27.65%; ventricle: 75.40 ± 19.41%) (**Figures 4A, D**) and CD105-positive (atrium: 89.30 ± 9.21%; ventricle: 95.67 ± 4.97%) (**Figures 4B, E**) cell populations. Fibroblasts were identified by vimentin expression with a purity greater than 95% (atrium: 95.53 ± 5.13%; ventricle: 96.17 ± 2.25%) (**Figures 4C, F**). Marker expression frequencies were comparable between the atrium and ventricle. Lentiviral vectors containing the sequence for a secreted NanoLuc as a reporter gene allowed assessment of transduction efficiency by measuring RLU in cell culture supernatants of ECs and fibroblasts. Bioluminescence levels increased continuously in the supernatants of cardiac ECs isolated from lentiviral vector-transduced hearts, reaching 2.82x10^6^ ± 2.69x10^6^ RLU (shNS; atrium), 3.56x10^6^ ± 4.94x10^6^ RLU (shNS; ventricle), 3.94x10^6^ ± 3.83x10^6^ RLU (shβ2m+CIITA; atrium), and 4.47x10^6^ ± 6.82x10^6^ RLU (shβ2m+CIITA; ventricle) after 8 days of cultivation (**Figure 4G**). Accordingly, increased NanoLuc activity was detected in the supernatants of cultured fibroblasts isolated from lentiviral vector-transduced hearts after 8 days, as indicated by 2.97x10^6^ ± 3.40x10^6^ RLU (shNS; atrium), 0.78x10^6^ ± 0.38x10^6^ RLU (shNS; ventricle), 3.89x10^6^ ± 2.90x10^6^ RLU (shβ2m+CIITA; atrium), and 0.83x10^6^ ± 0.38x10^6^ RLU (shβ2m+CIITA; ventricle). Bioluminescence activity remained at low levels of 1.07x10^2^ ± 0.34x10^2^ RLU (atrium) and 0.94x10^2^ ± 0.12x10^2^ RLU (ventricle) in the supernatants of fibroblasts isolated from non-TD hearts. Remarkably, RLU levels of fibroblasts isolated from the atrium of shβ2m+CIITA-transduced hearts were significantly increased (*p*<0.01) after 8 days of cultivation compared to fibroblasts isolated from the atrium and ventricle of non-TD hearts (**Figure 4H**). Therefore, normothermic EVHP enabled the generation of stable genetically engineered ECs and fibroblasts.

### 3.5 EVHP with lentiviral vectors allows stable transduction of cardiac tissues

**Figure 5:**
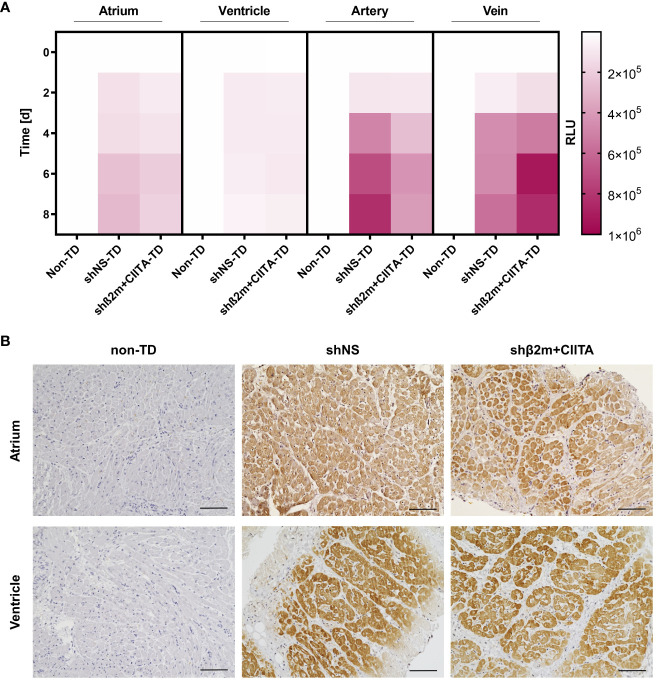
Efficiency of genetic engineering of cardiac tissues. **(A)** Heatmap represents NanoLuc activity of cardiac biopsies collected from the atrium and ventricle, coronary artery, and coronary vein of non-TD, shNS-transduced, and shβ2m+CIITA-transduced hearts. Bioluminescence activity of cultured tissues was determined by measuring RLU over a period of eight days. Color saturations represent means of NanoLuc activity. Statistical analysis was performed using two-way ANOVA. **(B)** Representative images show the localization of NeonGreen-expressing cardiac cells in the atrium and ventricle of non-TD, shNS-transduced, and shβ2m+CIITA-transduced hearts. Sections were stained with anti-NeonGreen antibody and counterstained with hematoxylin (scale bar: 100 µm).

In addition, transduction efficiency was evaluated by detecting NanoLuc activity in the supernatants of cultured biopsies from the atrium and ventricle, as well as cultured coronary arteries and coronary veins. Bioluminescence levels of 2.79x10^5^ ± 4.57x10^5^ RLU (shNS; atrium), 0.50x10^5^ ± 0.62x10^5^ RLU (shNS; ventricle), 1.81x10^5^ ± 1.32x10^5^ RLU (shβ2m+CIITA; atrium), and 0.63x10^5^ ± 0.53x10^5^ RLU (shβ2m+CIITA; ventricle) were measured in biopsy cultures of lentiviral vector-transduced hearts after 8 days. Similarly, the bioluminescence activity increased to 8.27x10^5^ ± 3.79x10^5^ RLU (shNS; coronary artery), 5.68x10^5^ ± 1.88x10^5^ RLU (shNS; coronary vein), 3.89x10^5^ ± 0.87x10^5^ RLU (shβ2m+CIITA; coronary artery), and 8.39x10^5^ ± 10.05x10^5^ RLU (shβ2m+CIITA; coronary vein) after 8 days of coronary vessel culture. Coronary vessels from non-TD hearts showed low levels of 0.71x10^2^ ± 0.19x10^2^ RLU (coronary artery) and 0.68x10^2^ ± 0.13x10^2^ RLU (coronary vein) (**Figure 5A**).

Furthermore, the distribution of genetically modified cardiac cells was determined in cultivated tissues from the atrium and ventricle after EVHP using lentiviral vector particles encoding for the NeonGreen reporter gene. Localization of reporter protein expression was evaluated by immunohistochemical staining of non-TD, shNS-transduced, and shβ2m+CIITA-transduced heart sections. Tissue samples from hearts perfused without lentiviral vector particles served as controls and showed no NeonGreen expression in the different heart regions. In contrast, a homogeneous distribution of NeonGreen-expressing cardiac cells was found in the atrium and ventricle of shNS-transduced and shβ2m+CIITA-transduced hearts (**Figure 5B**). Thus, normothermic EVHP using lentiviral vector particles allowed efficient transduction and stable transgene expression in cardiac tissues confirming previous observations at the cellular level.

### 3.6 EVHP with lentiviral vectors allows stable SLA class I and class II silencing in ECs and fibroblasts

Stable silencing of SLA class I and class II expression can reduce the strength of allogeneic immune responses by creating an immunologically invisible state of tissues and organs that supports graft survival (14, 23). Lentiviral vectors encoding for shRNAs targeting β2m and CIITA were delivered to the heart during EVHP. IFN-γ stimulation of ECs and fibroblasts mimicked a pro-inflammatory environment leading to upregulation of SLA class I and class II molecules.

**Figure 6:**
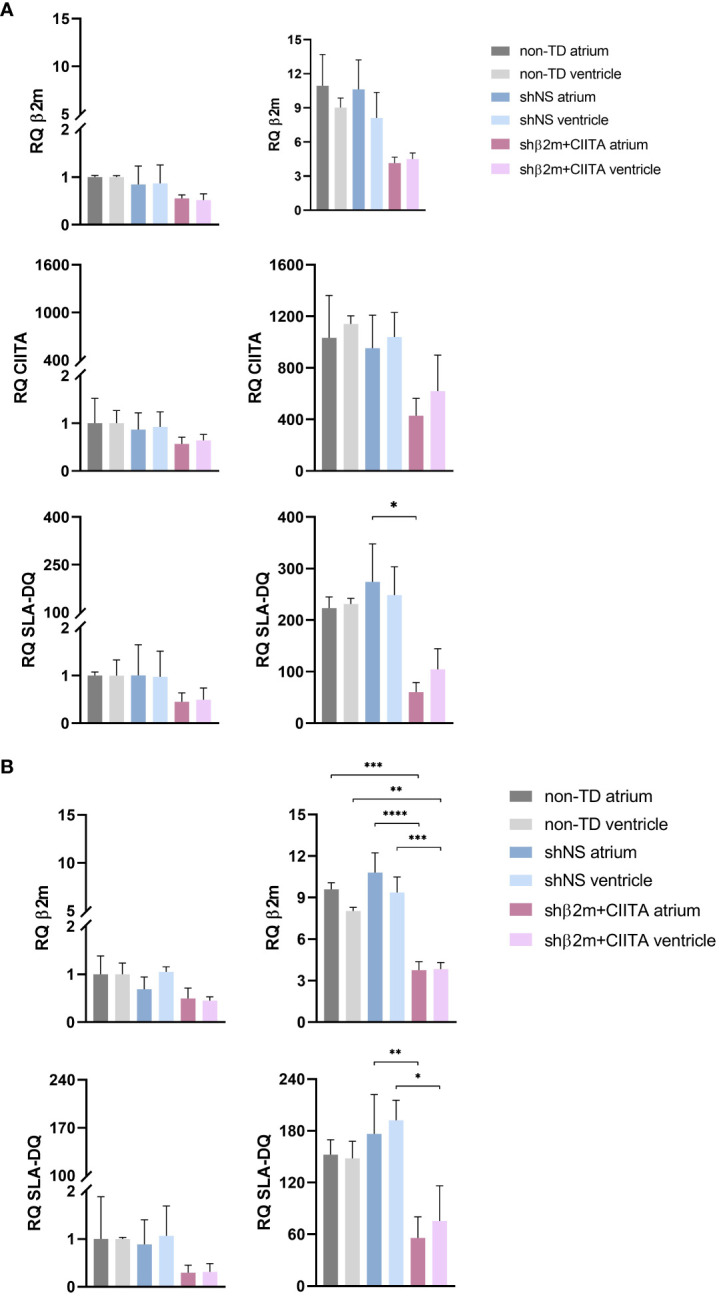
Silencing of SLA class I and class II gene expression in ECs and fibroblasts. **(A)** RQ of β2m, CIITA, and SLA-DQ transcript levels were detected in non-stimulated and IFN-γ-stimulated ECs **(A)** and fibroblasts **(B)** isolated from the atrium and ventricle of non-TD, shNS-transduced, and shβ2m+CIITA-transduced hearts after EVHP. Data were normalized to non-transduced tissue samples, which served as controls. Graphs represent mean and standard deviation. SLA class I and class II-related transcript levels were significantly decreased in stimulated ECs and fibroblasts from shβ2m+CIITA-transduced hearts (*p<0.05; **p<0.01; ***p<0.001; ****p<0.0001; one-way ANOVA).

**Figure 7:**
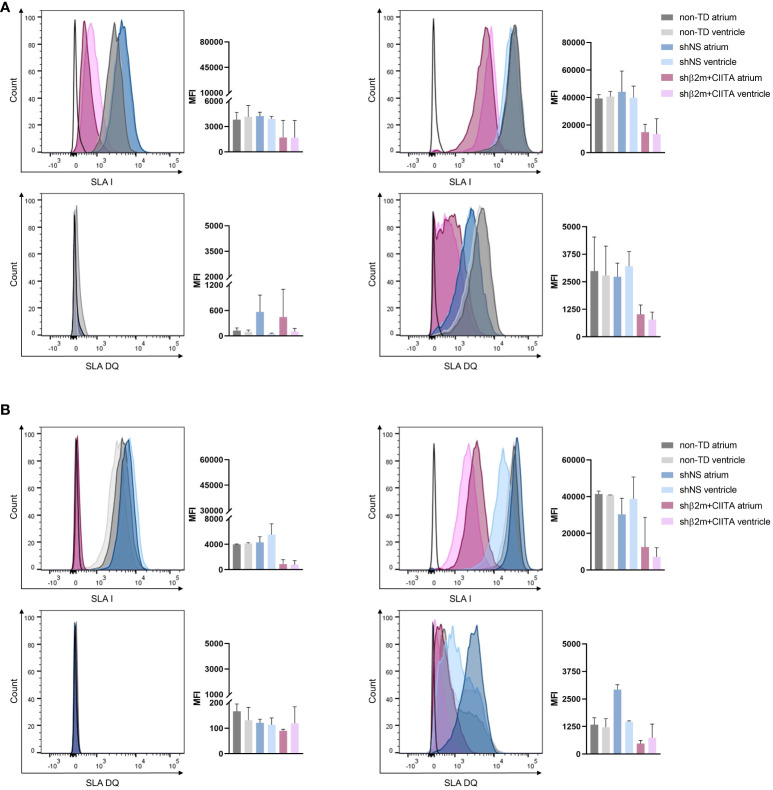
Silencing of SLA class I and class II protein expression in ECs and fibroblasts. Representative histogram plots and corresponding mean fluorescence intensity (MFI) graphs represent SLA class I and SLA-DQ expression in non-stimulated and IFN-γ-stimulated ECs **(A)** and fibroblasts **(B)** isolated from the atrium and ventricle of non-TD, shNS-transduced, and shβ2m+CIITA-transduced hearts after EVHP. Graphs represent mean and standard deviation. Statistical analysis was performed using one-way ANOVA.

Remarkably, silenced hearts showed up to 61% decreased SLA class I and up to 78% decreased SLA class II-DQ expression at the transcript level, resulting in a reduction of up to 66% and up to 76%, respectively, at the protein level in the vascular endothelium compared to shNS-transduced hearts. ECs isolated from shβ2m+CIITA-transduced hearts demonstrated an upregulation of β2m transcript levels by 4.14 ± 0.53-fold (atrium) and 4.49 ± 0.55-fold (ventricle) after IFN-γ stimulation compared to transcript levels of ECs isolated from shNS-transduced hearts by 10.63 ± 4.48-fold (atrium) and 8.12 ± 2.24-fold (ventricle) and transcript levels of ECs isolated from non-TD hearts by 10.95 ± 2.75-fold (atrium) and 9.02 ± 0.85-fold (ventricle) (**Figure 6A**). At the protein level, flow cytometry revealed a MFI of 14,897.33 ± 9,893.16 (atrium) and 13,492.67 ± 11,133.96 (ventricle) for ECs isolated from shβ2m+CIITA-transduced hearts, 44,165.33 ± 26,195.31 (atrium) and 39,863.33 ± 14,697.34 (ventricle) for ECs isolated from shNS-transduced hearts, and 39,394.50 ± 3,844.54 (atrium) and 40,786.50 ± 5,169.66 (ventricle) for ECs isolated from non-TD hearts (**Figure 7A**). In addition, ECs isolated from shβ2m+CIITA-transduced hearts only upregulated CIITA transcript levels by 429.02 ± 143.33-fold (atrium) and 619.91 ± 279.19-fold (ventricle), while ECs isolated from shNS-transduced hearts showed an increase of 952.51 ± 443.35-fold (atrium) and 1,039.79 ± 189.77-fold (ventricle) and ECs isolated from non-TD hearts of 1,032.90 ± 464.65-fold (atrium) and 1,140.92 ± 61.97-fold (ventricle). Accordingly, silencing of CIITA expression led to corresponding SLA-DQ transcript levels after IFN-γ stimulation, as demonstrated by upregulations of 60.25 ± 18.74-fold (atrium) and 104.57 ± 39.80-fold (ventricle) in ECs isolated from shβ2m+CIITA-transduced hearts compared to 274.17 ± 127.64-fold (atrium; *p*<0.05) and 248.38 ± 55.00-fold (ventricle) in ECs isolated from shNS-transduced hearts and 223.28 ± 21.41-fold (atrium) and 231.23 ± 10.90-fold (ventricle) in ECs isolated from non-TD hearts (**Figure 6A**). Notably, downregulation of SLA-DQ transcript levels in ECs resulted in a reduction of SLA-DQ protein expression, which was indicated by MFI values of 1,024.33 ± 736.48 (atrium) and 783.33 ± 590.27 (ventricle) for shβ2m+CIITA-transduced hearts compared to MFI values of 2,730.00 ± 1,073.29 (atrium) and 3,203.67 ± 1,149.20 (ventricle) for shNS-transduced hearts and 2,986.00 ± 2,184.96 (atrium) and 2,783.50 ± 1,887.27 (ventricle) for non-TD hearts (**Figure 7A**).

Similarly, silenced hearts showed up to 65% decreased SLA class I and up to 68% decreased SLA-DQ expression at the transcript level, resulting in a reduction of up to 82% and up to 84%, respectively, at the protein level in fibroblasts compared to shNS-transduced hearts. Fibroblasts isolated from shβ2m+CIITA-transduced hearts revealed a significantly lower upregulation of β2m transcript levels by 3.76 ± 0.61-fold (atrium) and 3.83 ± 0.48-fold (ventricle) after IFN-γ stimulation compared to an increase of 10.81 ± 1.42-fold (atrium; *p*<0.0001) and 9.37 ± 1.12-fold (ventricle; *p*<0.001) in fibroblasts isolated from shNS-transduced hearts and 9.60 ± 0.48-fold (atrium; *p*<0.001) and 8.04 ± 0.26-fold (ventricle; *p*<0.01) in fibroblasts isolated from non-TD hearts (**Figure 6B**). This contributed to a decrease in SLA class I protein expression, as measured by MFI values of 12,550.67 ± 16,187.35 (atrium) and 7,170.33 ± 8,750.78 (ventricle) for fibroblasts isolated from shβ2m+CIITA-transduced hearts compared to MFI values of 30,353.67 ± 15,063.75 (atrium) and 38,763.00 ± 20,653.86 (ventricle) for fibroblasts isolated from shNS-transduced hearts and 41,431.50 ± 2,249.31 (atrium) and 40,838.50 ± 136.47 (ventricle) for fibroblasts isolated from non-TD hearts (**Figure 7B**). Moreover, fibroblasts isolated from shβ2m+CIITA-transduced hearts indicated a significantly lower increase in SLA-DQ transcript levels by 55.98 ± 24.41-fold (atrium) and 75.56 ± 40.69-fold (ventricle) after IFN-γ stimulation compared to an upregulation of 176.43 ± 45.62-fold (atrium; *p*<0.01) and 192.24 ± 23.19-fold (ventricle; *p*<0.05) in fibroblasts isolated from shNS-transduced hearts and 152.43 ± 17.28-fold (atrium) and 148.04 ± 19.96-fold (ventricle) in fibroblasts isolated from non-TD hearts (**Figure 7A**). Remarkably, downregulation of SLA-DQ transcript levels in fibroblasts resulted in a reduction of SLA-DQ protein expression, as demonstrated by MFI values of 1,771.00 ± 2,237.52 (atrium) and 744.33 ± 617.37 (ventricle) for shβ2m+CIITA-transduced hearts compared to MFI values of 2,925.00 ± 377.11 (atrium; *p*<0.01) and 1,464.67 ± 68.72 (ventricle; *p*<0.05) for shNS-transduced hearts and 1,333.50 ± 444.77 (atrium) and 1,215.50 ± 548.01 (ventricle) for non-TD hearts (**Figure 7B**). Non-TD and shNS-transduced hearts showed comparable SLA class I and class II transcript and protein levels in ECs and fibroblasts. These data demonstrate the feasibility of silencing SLA class I and class II gene and protein expression in cardiac cells isolated from different heart regions after vector-mediated shRNA delivery.

## 4 Discussion

In this study, we have successfully established a platform for the stable genetic engineering of the heart during normothermic EVHP. Genetic engineering of the heart offers the possibility of correcting inherited genetic diseases that may cause heart failure, as well as having the potential to confer specific properties to the cardiac graft, that may protect it against rejection after allogeneic or xenogeneic transplantation. Stable expression of transgenes throughout the heart, including different cell subsets, is essential in both cases. Our group has been interested in genetically engineering grafts toward decreasing their immunogenicity by reducing major histocompatibility complex (MHC) expression (e.g., HLA in human tissues and SLA in porcine tissues).

*Ex vivo* organ perfusion has emerged as a powerful tool in the field of organ transplantation and genetic engineering. This innovative approach offers the potential to manipulate or optimize organ function prior to transplantation by creating the optimal environment for the delivery of gene therapeutic vectors (24). Our group has recently demonstrated the feasibility of genetically engineering various organs, including lung, kidney, and vascularized composites, using lentiviral vector particles (14–16).

Previously, several studies have reported successful cardiac transduction by delivery of adenoviral or AAV vectors to the heart during *ex vivo* machine perfusion (25, 26). However, these vector systems are susceptible to pre-existing immunity, which increases the risk of innate immune responses and tissue inflammation, thereby reducing transduction efficiency due to potential vector clearance (27). Additionally, adenoviral and AAV vectors are not capable of integrating the transgene into the host genome, leading to transient modifications. Consequently, lentiviral vectors are considered superior for the manipulation of genes that affect graft survival, as they provide long-term transgene expression and avoid the need for repeated vector administration (12).

The strategy of genetic engineering during normothermic EVHP may provide an efficient and robust platform for modulating non-immunological and immunological processes to prevent cardiac allograft loss. By introducing specific genes or gene modifications, protective pathways involved in cellular stress responses and antioxidant defense can be activated, thereby reducing the extent of ischemia-reperfusion injury, preserving organ function, and contributing to graft viability. A previous study showed attenuation of ischemia-reperfusion injury after superoxide dismutase gene transfer, responsible for neutralizing reactive oxygen species, into a donor heart during *ex vivo* organ preservation (28). Genetic modification can further stimulate tissue repair and regeneration within the graft by delivering genes that enhance cell proliferation, angiogenesis, and tissue remodeling (29). Instead of manipulating the recipient’s immune system by using immunosuppression, which increases susceptibility to opportunistic infections and tumors, *ex vivo* machine perfusion (EVMP) may allow targeted modification of the donor organ toward reducing graft immunogenicity. For this purpose, gene therapy can be used to intervene in various targets of the immune response, such as TLR signaling or T-cell pathways. Previously, blockade of the CD28/B7 costimulatory T-cell pathway has been shown to promote cardiac allograft survival and donor- and organ-specific tolerance in different animal models (30, 31).

Lentiviral vectors are an option for achieving sustained gene expression in a variety of cell types present in an organ. Previous studies have reported high lentiviral vector-mediated transduction rates at 37°C (32, 33). VSV-G protein binds to the LDL receptor of target cells at temperatures of 4°C, but temperatures around 37°C are required for lentivirus uptake (34). In addition, the temperature range in which transduction efficiency is most efficient also depends on the thermostability of the lentiviral particles. Lentiviral vectors exhibit a half-life of 7–8 hours at 37°C due to their complexity (35).

In this study, we showed the feasibility of genetically engineering porcine hearts with lentiviral vectors during normothermic EVHP by measuring a sustained expression of the NanoLuc reporter gene in vascular endothelial cells and fibroblasts isolated from the heart. Similar results were found in cultivated cardiac tissues. Although we detected different levels of NanoLuc expression in the cultured vessels and biopsies, all regions of the heart were genetically modified. In particular, coronary arteries and veins directly exposed to lentiviral vector particles demonstrated a higher NanoLuc expression than cardiac biopsies isolated from the atrium and ventricle. However, immunohistological analysis confirmed a homogeneous distribution of NeonGreen-expressing cells in all cardiac regions of lentiviral vector-transduced hearts. The vascular endothelium represents the first barrier between the allograft and the recipient’s immune system and therefore plays an essential role in coordinating innate and adaptive donor-specific immune responses (36). Consequently, the organ endothelium can be considered the most relevant target for genetic engineering, aiming at improving graft survival.

Hence, this study investigated the potential of transducing hearts with lentiviral vector particles, encoding for specific shRNAs targeting β2m and CIITA, to permanently reduce SLA class I and class II expression at the transcriptional and protein levels. We have previously shown that silencing MHC expression in cells and simple tissues results in the prevention of *de novo* immune responses and protection of allogeneic cells from pre-formed humoral immunity *in vivo* (37–39). This approach may represent a promising tool to specifically suppress humoral and cellular immune responses in allogeneic and xenogeneic transplantation settings.

We used IFN‐γ to mimic an inflammatory environment that increases SLA class I expression and stimulates SLA class II expression (40). Even under the IFN‐γ-mediated inflammatory environment, we observed significantly decreased expression of SLA class I and class II transcripts and proteins in endothelial cells and fibroblasts isolated from genetically modified porcine hearts. Downregulation of SLA expression by RNA interference has several advantages over a complete gene knockout mediated by CRISPR/Cas9 technology. The absence of MHC class I molecules would induce alloreactive NK cell activation, resulting in tissue damage due to increased NK cell-mediated cytotoxicity (39). Thus, RNA interference technology offers a promising strategy to permanently reduce organ immunogenicity by targeting MHC class I and class II expression without causing unwanted immune reactions.

In the past, *ex vivo* machine perfusion has been described as a re-conditioning strategy for maintaining or even improving organ quality and integrity prior to transplantation (41). Lactate levels are considered an important prognostic marker for cardiac dysfunction (42, 43). Since the heart can consume lactate independently, decreasing lactate levels would indicate a functioning metabolism (44). The slightly elevated lactate levels in the perfusate may be attributed to a natural stress response of the heart and the absence of homeostatic organs such as the liver and lungs. Moreover, a similar increase in lactate levels has been found in other perfusion models without affecting transplant outcome (45). Additional markers predicting cardiac viability include troponin T and LDH. While LDH serves as a general cell and tissue damage marker, high levels of troponin T primarily indicate the presence of muscle injury and acute myocardial infarction (19). As previously demonstrated in various porcine EVMP models, LDH and troponin T are released from injured cardiomyocytes and accumulate in the perfusion circulation (46, 47). Consistent with these findings, we detected a slight increase in LDH and troponin T levels during EVHP, which could be explained as a consequence of the artificial perfusion setting using different pumps and the short period of cold ischemia prior to perfusion (16, 48). Importantly, lentiviral vector delivery did not contribute to additional tissue damage, as shown by comparable lactate, LDH, and troponin T levels. In addition to the analyzed tissue injury markers, a comprehensive evaluation of cardiac function during EVHP is highly desirable and should be performed in future studies. As already reported in previous studies with rodents, a balloon filled with saline solution can be placed in the left ventricle to measure the left ventricular diastolic pressure in real-time (49–51). In future studies, this method may also be suitable for the assessment of heart function during EVHP.

*Ex vivo* machine perfusion has been reported to mobilize immune cells and stimulate the release of pro-inflammatory mediators that may affect allogeneic immune responses (52, 53). During EVHP, we observed elevated IL-6, IL-8, and TNF-α secretion, which may indicate endothelium activation. The formation of IL-6 is related to early incipient myocardial ischemia and contact of cells with artificial and non-endothelialized surfaces, whereas IL-8 production is mainly explained by the shear stress of the perfusion system. TNF-α production is further stimulated by high levels of IL-6 (54). Simultaneous upregulation of adhesion molecules (ICAM-1 and V-CAM-1) during EVMP promotes leukocyte-endothelial interactions and subsequent immune cell infiltration into cardiac tissue (21). The increase in IL-6, IL-8, and TNF-α levels has already been described in several studies and showed a similar course to cardiopulmonary bypass or extracorporeal membrane oxygenation (54, 55). However, several strategies, including hemoadsorbers and pharmacological drugs, are currently being developed to minimize cytokine production that could lead to immune polarization after HTx (56, 57). Recently, machine perfusion was identified to induce the activation of protective repair mechanisms, potentially mitigating the damage caused by ischemia-reperfusion injury. Upregulation of HSP70 expression counteracts myocardial tissue injury under EVHP conditions by decreasing the development of inflammatory processes and contributing to tolerance (58, 59). HIF-1α has been implicated in the regulation of angiogenesis and remodeling after reduced cardiac perfusion by inducing vascular endothelial growth factor and nitric oxide synthase production (60). In this study, HIF-1α expression was not significantly increased, indicating adequate tissue oxygenation during acellular perfusion.

In addition to its potential use in allotransplantation settings, this technology may also bring several benefits in the field of xenotransplantation, as it may allow the correction of genes that have a detrimental or even lethal effect during embryonal or pig development. The first pig-to-human heart transplant in January 2022 marked a milestone that brought xenotransplantation one step closer to clinical practice (61). The use of porcine hearts offers an opportunity to alleviate the shortage of human organs with an unlimited supply of organs due to their physiological and genetic similarity to human hearts (62). However, genetic differences in cell surface proteins or carbohydrates exist and are associated with immunological hurdles. For example, cross-reaction of anti-HLA antibodies with SLA may increase the risk of rejection (63). In the field of xenotransplantation, genetic modification also provides a promising approach to reduce the increased risk of rejection caused by genetic variations of different species. Specific gene knock-out pigs often exhibit significant deficiencies in the performance of the immune system and are usually not capable of surviving outside of the appropriate breeding facilities. *Ex vivo* engineering offers the possibility of optimally adapting the organ to the recipient (64).

This strategy allows the *ex vivo* reduction of MHC class I and II molecules, which is a promising approach to reduce the immune responses after transplantation in allogeneic and xenogeneic settings and thus may contribute to prolonged organ survival (23, 65). However, further transplantation studies are required to monitor the effects of SLA knockdown in allogeneic and xenogeneic immune responses.

This study demonstrates the feasibility of permanent genetic engineering of the heart during *ex vivo* perfusion without compromising tissue integrity. The use of lentiviral vector particles allows long-term modification of gene expression. The short window between organ procurement and transplantation enables *ex vivo* organ engineering, thereby avoiding systemic adverse effects associated with *in vivo* gene therapy. In the field of allotransplantation, genetic engineering offers a unique opportunity to modify genes related to ischemia-reperfusion injury, graft immunogenicity, or tissue regeneration. In particular, we were able to show downregulation of SLA class I and II expression in different cardiac cell types. The generation of immunologically invisible allografts provides the potential to reduce cellular and antibody-mediated rejection, which may support graft survival after allogeneic or xenogeneic HTx and combat the burden of immunosuppressive therapy.

## Data availability statement

The original contributions presented in the study are included in the article. Further inquiries can be directed to the corresponding author.

## Ethics statement

The animal study was approved by LAVES-Niedersächsisches Landesamt für Verbraucherschutz und Lebensmittelsicherheit. The study was conducted in accordance with the local legislation and institutional requirements.

## Author contributions

KS: Conceptualization, Formal analysis, Data curation, Investigation, Methodology, Visualization, Writing – original draft. TR: Formal analysis, Investigation, Methodology, Visualization, Writing – original draft. JB: Investigation, Methodology, Visualization, Writing – review & editing. HV: Data curation, Investigation, Writing – review & editing. KH: Data curation, Investigation, Writing – review & editing. GD: Investigation, Writing – review & editing. AR: Resources, Writing – review & editing. JS: Investigation, Writing – review & editing. RB: Conceptualization, Resources, Writing – review & editing. CF: Conceptualization, Formal analysis, Funding acquisition, Investigation, Methodology, Project administration, Supervision, Writing – original draft, Writing – review & editing.
